# Experiences of Working With Refugee Children From Ukraine: An Interview Study With School Nurses

**DOI:** 10.1111/phn.70075

**Published:** 2026-01-23

**Authors:** Pernille Korf, Pernilla Garmy, Eva‐Lena Einberg

**Affiliations:** ^1^ Department of Nursing and Health Sciences Faculty of Health Sciences Kristianstad University Kristianstad Sweden; ^2^ Department of Health Sciences Faculty of Medicine Lund University Lund Sweden

**Keywords:** experience, health promotion, mental health, refugee children, school nurse, Ukraine, war

## Abstract

**Objective:**

The aim of this study was to investigate the experiences of school nurses working with refugee children from Ukraine.

**Design:**

This research was conducted as a qualitative interview study, using an inductive approach and a semi‐structured interview guide.

**Sample:**

School nurses (*n* = 8) from different parts of Sweden were interviewed, and the data were analyzed through a qualitative content analysis. The COREQ guidelines were followed for reporting study findings.

**Results:**

Four categories were identified in the analysis: (1) Initial health conversations with the Ukrainian refugee children, (2) Experience of challenges during the health visits, (3) The impact of war on the Ukrainian refugee children, and (4) To have a health‐promoting everyday life.

**Conclusion:**

The present study shows that the school nurse has an important function in promoting the health of Ukrainian refugee children, and describes the health conversation as a significant part of the meeting with these children. The work of public health nurses in schools in supporting Ukrainian refugee children can be optimized through increased cultural awareness, which can help promote the children's overall health.

**Patient or Public Contribution:**

The study question was identified in discussion with school nurses.

## 1 Introduction

The sudden influx of Ukrainian refugees has created a unique situation in Europe, including Sweden. This has affected the daily work of school nurses, as refugee children present with various physical and psychological challenges (Abdi et al. 2023; Hjern and Kling 2019; Ludvigsson and Loboda 2022). School nurses play a crucial role in promoting health and preventing illness among refugee children (Hjern et al. 2019; Musliu et al. 2019). However, there is a lack of studies specifically focusing on school nurses’ work with Ukrainian refugee children. Addressing this gap is important for contributing new knowledge to healthcare and school health services.

### 1.1 Background

Following Russia's full‐scale invasion of Ukraine in 2022, approximately 6.3 million people have fled the country as refugees or asylum‐seekers, nearly 6 million of whom are in Europe. Women and children constitute 76% of these refugees (UNHCR 2023). Most have sought refuge in neighboring European countries, with Sweden receiving over 50,000 Ukrainian refugees, many of them school‐aged children. Most Ukrainian refugees in Sweden have applied for and received residence permits, allowing them to work and attend school (Migration Agency 2023). The Mass Migration Directive ensures that Ukrainian children have the same access to education and healthcare as Swedish children, bringing these children into contact with school nurses (National Board of Health and Welfare 2023). This refugee situation has placed new demands on school health services, particularly in identifying health needs and promoting well‐being among children who have experienced trauma, loss, and displacement.

In Sweden, education is publicly funded and free of charge for all children and adolescents. Schools may be public or private, but all follow the national education framework. According to the Swedish Education Act (2010), every school must have access to a school nurse as part of the school health service. The school nurse's responsibilities include conducting routine health visits with all students, providing vaccinations according to the national immunization program, and offering drop‐in health consultations. In addition, school nurses work with health promotion at both individual and group levels.

The war in Ukraine and the influx of refugees encountered by nurses in host countries present ethical dilemmas and moral challenges in nurses' daily work. These circumstances necessitate reflection on the International Council of Nurses' (ICN) Code of Ethics (2021). Operationalizing the ICN Code of Ethics requires ethical reflection and discussion across all contexts in which nurses work, from policy‐making levels to direct care environments (Lindberg and Brinchmann 2023). A cross‐sectional study from Ireland found that although public health nurses were highly aware of the importance of culturally sensitive care, implementing such care in practice was challenging (Oakley et al. 2024).

Uncertainty was identified as a main theme in a qualitative meta‐synthesis on migrant health (Villar‐Bustos et al. 2024), alongside interrelated themes such as language, social networks, and employment. Among asylum‐seeking families, there is an increased risk of health problems (Blackmore et al. 2019; Kien et al. 2019). A Turkish study of asylum seekers from Afghanistan shows that children's growth and development are significantly negatively affected (Bulucu Büyüksoy 2023). Children experience three stages of fleeing: life before fleeing, the journey, and adjusting to a new country, each causing stress and potential post‐traumatic stress disorder (PTSD) (Measham et al. 2014). The Health Care Act in Sweden mandates equal healthcare for all, including free health examinations for asylum seekers (Migration Agency 2023). The Education Act in Sweden (2010) requires access for all to comprehensive school health services, including medical, psychological, psychosocial, and special educational support.

Measham et al. (2014) emphasize the value of supporting psychological well‐being and positive adaptation following migration. Larson et al. (2024) highlight the importance of developing intercultural nursing care for refugees from Ukraine. A 2019 study highlighted the school nurse's role in supporting unaccompanied refugee children primarily from Syria and Afghanistan, emphasizing the need for knowledge of PTSD, intercultural understanding, and self‐awareness (Musliu et al. 2019). Wahlström's (2022) doctoral thesis, “The relevance of cultures,” explores how Swedish school nurses conduct health visits with children who have migration backgrounds. The study shows that nurses adapt these visits based on the child's background, often relying on non‐verbal communication due to language barriers. Effective communication and cultural sensitivity are identified as key to successful encounters, underscoring the importance of school nurses’ responsiveness and cultural awareness. To develop school nurses' supportive interventions for children who have fled their home countries, knowledge of the school nurses' experiences of meeting these children is required. Thus, the purpose of this study was to investigate school nurses' experiences of working with refugee children from Ukraine.

## 2 Methods

### 2.1 Research Design

A qualitative descriptive design was used. This approach is suitable for investigating clinically relevant topics, such as school nurses’ experiences of working with refugee children, while remaining closely aligned with the data (Sandelowski 2010). The study followed the consolidated criteria for reporting qualitative research (COREQ) (Tong et al. 2007).

### 2.2 Sample and Setting

The study was conducted among school nurses in Sweden who worked with children and adolescents aged from 6 to 19 years, see Table 1. The school nurses worked in both public and private schools.

**TABLE 1:** Sample characteristics.

Interview	Age (years)	Specialist education	Experience as school nurse (years)	School setting (student age)
A	41	Public health	3	Upper secondary (ages 16–19)
B	46	Public health	5	Junior secondary (ages 13–15)
C	43	Public health	5	Primary and middle (ages 6–12)
D	38	Public health	1	Primary to junior secondary (ages 6–15)
E	39	Child and adolescent health	10	Primary to junior secondary (ages 6–15)
F	50	Public health	3	Upper secondary (ages 16–19)
G	30	Child and adolescent health	4	Primary to junior secondary (ages 6–15)
H	61	School health	2	Upper secondary (ages 16–19)

*Note*: All participants were female registered nurses with Swedish postgraduate specialist education and were employed as school nurses. In Sweden, the role of school nurse requires postgraduate education in one of three areas: public health nursing (district nursing), child and adolescent health (pediatric nursing), or school health (school nursing).

Purposeful sampling was conducted. The inclusion criterion was school nurses in Sweden with experience of working with refugee children from Ukraine in their work during 2022–2023. The first author contacted (with e‐mail and one reminder) 80 school leaders in municipalities that had received Ukrainian refugees. Of these, 33 school leaders declined participation due to a lack of Ukrainian students or a lack of time. No response came from 34 schools. In 13 schools, the principals gave approval to the study, and among these schools, eight school nurses agreed to participate in the study. We did not ask for reasons from the five school nurses who declined participation.

All participants were female, aged between 30 and 61 years (median age 42). All were registered nurses with Swedish postgraduate specialist education in either public health nursing (*n* = 5), child and adolescent health nursing (*n* = 2), or school health nursing (*n* = 1), qualifying them to work as school nurses.

### 2.3 Data Collection

Data were collected through semi‐structured interviews conducted by the first author; a female registered nurse enrolled in a Master's program in public health nursing (district nursing). The interview guide (Appendix 1) included open‐ended questions such as: “What is your experience of meeting refugee children from Ukraine?” and “Can you tell us about a particular situation?”

A pilot interview was conducted to test the relevance and clarity of the interview guide; as no revisions were deemed necessary, this interview was included in the sample. Seven interviews were conducted via Zoom, and one interview was conducted by telephone due to technical problems with the participant's computer. All interviews were audio‐recorded and lasted approximately 45 min.

The audio recordings were transcribed verbatim by the first author. The transcriptions were stored on a secure, password‐protected server accessible only to the research team.

### 2.4 Data Analysis and Synthesis

Qualitative content analysis was used to analyze the interview data, following the approach described by Graneheim and Lundman (2004). In this study, their framework guided the entire analytical process, from identifying meaning units and coding to developing categories and themes.

After all interviews had been completed, the audio recordings were transcribed verbatim, and the full set of transcriptions constituted the unit of analysis. To gain an overall understanding of the material, all authors read the transcripts several times. The first author then identified meaning units related to the aim of the study. Meaning units were defined as segments of text that were relevant to and informative for the study's purpose. These meaning units were condensed into shorter formulations while preserving their core meaning, and the condensed meaning units were assigned codes that captured their content (Graneheim and Lundman 2004).

The initial coding was conducted manually by the first author and subsequently reviewed together with the third author. Codes were compared, discussed, and revised in an iterative process. Codes with similar content were grouped into preliminary subcategories, which were then further examined and abstracted into broader categories. At a later stage of the analysis, the subcategories were phased out, and only the four final categories were retained.

The analysis primarily focused on the manifest content by staying close to the participants’ descriptions, but it also included a latent level of interpretation, in which underlying meanings and patterns across the categories were considered. All three authors participated in discussions of the emerging categories and their interpretations until consensus was reached. Preliminary interpretations were also presented and discussed in research seminars with colleagues, which contributed to refining the analysis. No qualitative data analysis software was used; the coding and categorization were conducted entirely manually.

### 2.5 Rigor and Trustworthiness

Criteria for trustworthiness in qualitative research include credibility, dependability, confirmability, and transferability, as described by Lincoln and Guba (1985).

Credibility in the present study was strengthened through the inclusion of participants’ quotes in the findings, illustrating how interpretations were grounded in the data. Credibility was also enhanced by providing a clear and believable description of the study context.

Dependability was strengthened through the semi‐structured interview guide, with all participants being asked the same questions. A pilot interview was conducted to ensure the relevance of the interview questions, and no modifications were deemed necessary. Dependability was further supported by the use of the qualitative content analysis framework described by Graneheim and Lundman (2004). To enhance dependability, the authors strived to maintain neutrality throughout the analytical process.

Confirmability was addressed through the inclusion of participants from different parts of Sweden. All three authors have professional experience as registered nurses and have encountered refugee families in vulnerable situations. The second and third authors have each worked as school nurses for over ten years, providing extensive experience in working with refugee children and adolescents. While this professional background contributed to contextual understanding, it also required continuous reflexivity to minimize potential bias. Throughout the analysis, the research team engaged in regular discussions to critically examine interpretations, and preliminary findings were presented at research seminars with colleagues to enhance rigor and trustworthiness.

Transferability was supported through detailed descriptions of the study context, participant selection, data collection, and analysis process. The results may be transferable to similar contexts, particularly within European school health systems.

### 2.6 Ethical Considerations

The study was approved by the Swedish Ethical Review Authority prior to data collection (Reference number 2018–842; 2023‐01448‐02). All procedures followed the ethical principles for research involving human subjects outlined in the Declaration of Helsinki (WMA 2024). Written informed consent was obtained from all participants before participation.

### 2.7 Findings

Four categories were identified in the analysis: (1) Initial health conversations with the Ukrainian refugee children, (2) Experience of challenges during the health visits, (3) The impact of war on the Ukrainian refugee children, and (4) To have a health‐promoting everyday life.

### 2.8 Initial Health Conversations With the Ukrainian Refugee Children

School nurses shared experiences of initial health conversations with newly arrived Ukrainian refugee children and their parents. These meetings, often occurring on the second day of school, involved mixed emotions. One school nurse described how parents mentioned that health checks had already been done in Ukraine, but still found the Swedish meetings positive. Emotional reactions were common, especially when discussing the war.

“All the new arrivals meet me for a health conversation so I, it's right on the second day of school, I meet these students, and there they are with their parents.” (Interview B)

One school nurse noted that children in Ukraine do not receive certain vaccinations, such as the vaccine against human papillomavirus (HPV). Some school nurses found it challenging to confirm vaccination status due to missing records.

“It's been a bit troublesome… they don't have their vaccination cards…. and then you say, I think they followed the program, but we don't know.” (Interview G)

Accessibility was key, with school nurses emphasizing an open‐door policy to support Ukrainian refugee children, ensuring they felt welcome to discuss difficulties or seek help. Being present in various school settings provided a sense of safety.

“Otherwise, it's about being a safe adult… if they feel that they can always come here and talk if it would be something.” (Interview G)

Collaboration with other school professionals was described as essential for creating the conditions necessary for meaningful health conversations. School nurses emphasized the value of joint meetings with principals, teachers, interpreters, children, and guardians to establish a shared understanding of the school environment and routines. These meetings provided important contextual information, addressed practical concerns (such as school meals), and helped build trust between the school and the family—all of which facilitated more open and responsive health conversations.

“First, in a joint meeting with the principal, teacher, interpreter, the child, and guardians we kind of talk about the school, about how the school situation will look like, we show what kind of food is served in the school, and we kind of do that with everyone.” (Interview B)

### 2.9 Experience of Challenges During the Health Visits

School nurses described various challenges during health visits with Ukrainian refugee children, most notably related to communication. In many cases, the availability of professional interpreters was limited. Sometimes, teachers were asked to interpret, which required confidentiality agreements and occasionally led to problematic situations. For spontaneous visits, communication often relied on English or digital translation tools, which the school nurses felt were inadequate for more sensitive topics.

“We did not get any interpreters. Then the school had to ask the home language teachers to act as interpreters and they had to sign a confidentiality agreement. It turned out really bad.” (Interview G)

Moreover, time constraints limited the school nurses’ ability to address mental health concerns in depth. Some school nurses expressed feelings of inadequacy, particularly when they sensed unmet psychological needs among the children.

“As a school nurse, I can sometimes feel that their mental well‐being is lacking and I cannot do enough.” (Interview D)

Cultural differences also made it difficult to discuss psychological well‐being. School nurses noted that some parents did not acknowledge mental illness and tended to express psychological distress as physical symptoms.

“There is also this thing that you don't really want to talk about what is difficult. You just don't want to problematize so much around it, then I understand that there is a different culture among my other students from the Balkans and Eastern Europe; they don't believe in mental illness, they don't believe in anxiety or depression… but then it becomes more like you have a physical illness.” (Interview A)

Finally, school nurses observed that some children preferred to rely on family members or Ukrainian peers when dealing with emotional challenges. Cultural and emotional reactions were also evident in responses to topics such as sexual education.

“They themselves have felt that they have… they have their family to talk to. And they have their Ukrainian peers to talk to.” (Interview G)

“I've been out doing sexual education with the 12‐year‐olds and they've been completely shocked when… talking and showing films…. ‘What is this?’” (Interview D)

### 2.10 The Impact of the War on the Ukrainian Refugee Children

School nurses described how the war in Ukraine affected the emotional well‐being of refugee children. School nurses reported that many children expressed worry, sadness, and a sense of loss, particularly because fathers or older siblings had remained in Ukraine. This anxiety sometimes led to sleep difficulties or emotional withdrawal. Some families, including the father, were able to arrive in Sweden together before the border closure, while others had limited contact with family members who remained in Ukraine, which intensified the children's feelings of loneliness and worry.

Several school nurses also observed that children were affected by their mothers’ emotional states, often describing mothers as highly anxious or distressed. The school nurses reported that this dynamic sometimes led children to feel responsible for their mothers’ well‐being.

“They have a mother who is very, very worried, almost to the point that they feel they have to take care of her.” (Interview A)

The physical health of Ukrainian children was generally reported as good, with some exceptions requiring specialist care. Dental health problems were noted by some school nurses, but most children were considered physically healthy. However, psychological symptoms were prominent, especially among teenagers. Depressive symptoms, fatigue, headaches, and increased absenteeism were frequently reported. Some school nurses found these youths emotionally distant and difficult to reach.

“And when I see them, they tense up, you can see it when they walk… and they're not happy so I understand they have a headache.” (Interview D)

Several school nurses mentioned that many Ukrainian children attended school in both Sweden and Ukraine, which created long and stressful days. This double schooling was described as exhausting, particularly for older students.

“Some of them go to school in parallel when they go here, because they also go to school in Ukraine over the internet.” (Interview F)

Adapting to the Swedish school system also presented challenges. School nurses noted that Ukrainian children were not used to the Swedish model of group work, discussions, and individual support, in contrast to the stricter educational structure in Ukraine. Despite these challenges, the children were generally seen as motivated and capable students.

“Here in Sweden you can ask for help. It is much stricter in Ukraine, like it used to be in Sweden.” (Interview D)

### 2.11 To Have a Health‐Promoting Everyday Life

Several school nurses noted that Ukrainian refugee children of different age groups settled well in Sweden, appearing calm, safe, and well‐functioning. However, there was some variation in experiences. Some school nurses observed that Ukrainian refugee children had a strong drive, aiming to complete their education in Sweden and potentially return to Ukraine. Others found these children harder to motivate because they wanted to go back to Ukraine.

“There is some sort of confidence in them, such as they are thinking that they are here now and they feel they have to make the best of the present situation. Either they continue to study online with the Ukrainian curriculum, thinking that they will go back to Ukraine then, when the war is over, or that they are going to continue their education in Sweden because they are here now.” (Interview A)

Many school nurses noted that the children benefited from close‐knit peer groups with other Ukrainian students, which provided emotional support and reduced feelings of loneliness. However, some children remained isolated even within these communities, especially when few peers shared their background. The school nurses found that younger children generally found it easier to make friends and adjust socially.

“We have a girl here who was quite lonely and she was the only girl we had from Ukraine and then another girl from Ukraine came and then you kind of noticed how she blossomed. Because then there were two of them… how much easier it is.” (Interview F)

Language acquisition was seen as essential for integration. School nurses highlighted that younger children learned Swedish more easily through play and interaction, while older children often relied on English or digital tools. English proficiency helped some children adapt more quickly in the school setting.

“You can play hide and seek in any language.” (Interview D)

“So far they aren't so good at Swedish, but some of them are quite good at English and it works much easier then.” (Interview F)

Access to meaningful activities, both in and outside school, was viewed as critical for promoting well‐being. Younger children were often more engaged in school‐based activities, while older children—especially teenage girls—sometimes struggled to find suitable outlets and spent more time on their phones. Boys were more frequently reported to take part in organized sports or hobbies. Some nurses actively worked to connect children with extracurricular activities and aid organizations based on their interests.

“What I have tried and what I do as a school nurse when I meet them, is to try to help so that they can continue with sports and physical activity. Do they like music, play an instrument, and are they still able to continue with it? Some kind of normality in everything that they have been through.” (Interview C)

Creating a safe and welcoming school environment was another key aspect. Suggestions included starting in smaller classes to reduce stress and facilitate adjustment. One school nurse described how her school prepared for new arrivals by informing other students and decorating classrooms to create a warm atmosphere.

“After all, they had their own classroom where everyone gathered to begin with. There we tried to do something with sunflowers and such to make a nice environment.” (Interview C)

## 3 Discussion

This study explored school nurses’ experiences of working with Ukrainian refugee children. Overall, the nurses described how these children carried emotional burdens related to family separation, war‐related trauma, and uncertainty about the future. Rather than focusing solely on the expression of distress, the nurses’ accounts also revealed deeper interpretative patterns reflecting their efforts to work with a health‐promoting approach.

Our findings align with a report by Save the Children Denmark (2023), which highlights persistent loneliness and stress among Ukrainian children who simultaneously attend school in the host country and follow online education from Ukraine. In our study, school nurses perceived this dual schooling as a source of strain, particularly for adolescents who struggled to balance academic expectations from two systems. This may also reflect the children's strong desire to maintain continuity and identity with their home country, even while adapting to a new context.

The nurses described challenges in addressing psychological concerns, often due to cultural stigma surrounding mental health. Parents were described as reluctant to acknowledge mental illness, interpreting symptoms such as anxiety or depression as physical complaints. These finding echoes previous research indicating that cultural beliefs shape both symptom expression and help‐seeking behavior (Kuntz 2022). This may illustrate how school nurses act as mediators between healthcare norms in Sweden and the cultural frames of newly arrived families. As Wahlström et al. (2019) emphasize, this requires reflexivity, cultural sensitivity, and the ability to respond to unspoken needs.

Family separation has been identified in earlier studies as a major factor influencing the well‐being of refugee children (Bürgin et al. 2022; Waddoups et al. 2019). The school nurses in our study observed that many children, particularly adolescent girls, were emotionally affected by the ongoing war, showing signs such as fatigue, sadness, and absenteeism. These patterns correspond to findings from Catani et al. (2023), where nearly half of Ukrainian refugee children met criteria for PTSD. Our findings suggest that similar screening and early support mechanisms could be relevant in the Swedish context.

The school nurses emphasized the importance of observing behavioral and physical signs, such as fatigue, headaches, or mood changes, rather than directly asking about traumatic experiences. This approach aligns with the Swiss guidelines on supporting Ukrainian refugee children (Jaeger et al. 2022) and illustrates a trauma‐sensitive way of working. This reflects a caring strategy rooted in respect and caution, where the nurse protects the child from potential retraumatization while still remaining attentive to signs of distress.

Age differences among the children were also reflected in the nurses’ experiences. Younger children were perceived as more dependent on routines, parental presence, and structured support, whereas adolescents often faced challenges related to identity, autonomy, and belonging. Recognizing these developmental differences is essential for tailoring school health interventions and highlights the importance of flexibility in the school nurse's role.

Supporting mental health is central to children's overall well‐being and aligns with the UN Sustainable Development Goals (UNICEF 2023). The nurses in this study described how building trust, being available, and maintaining open communication were key strategies to help children feel safe. Their proactive efforts to create welcoming environments and promote participation in social and recreational activities illustrate the school nurse's preventive and health‐promoting role.

Visibility and accessibility were also crucial elements in building relationships. Several nurses described strategies to ensure that Ukrainian refugee children knew where and how to reach them. This finding supports previous research highlighting the importance of a visible and proactive school health service in reaching vulnerable groups (Bartlett 2015; Jönsson et al. 2019). Visibility can also be understood as a symbolic gesture of stability and safety, an embodied assurance that support is both present and approachable.

### 3.1 Limitations

Although 80 school leaders were contacted through purposive sampling, only eight school nurses ultimately chose to participate. The difficulty in recruiting participants may reflect the demanding and stressful nature of school nurses’ daily work; however, this also represents a limitation in terms of confirmability.

The data collection relied on school nurses’ experiences. Although this constitutes a valuable source of professional knowledge, the inclusion of interviews with children and parents who have fled war and resettled in Sweden could have enriched the findings.

## 4 Conclusions

This study contributes to a deeper understanding of how school nurses experience and navigate their work with Ukrainian refugee children in Swedish schools. The findings highlight the complex interplay of emotional, cultural, and structural factors that shape these encounters. Initial health conversations served as important entry points for identifying both physical and psychological needs, but language barriers and cultural stigma around mental illness posed significant challenges. School nurses emphasized the importance of being present, trustworthy, and responsive adults, particularly in situations where children were affected by family separation, trauma, and displacement.

Despite limited resources, the school nurses described strategies to support children's adaptation to a new environment, including facilitating peer connections, encouraging participation in meaningful activities, and promoting language development. Younger children were often more easily integrated, while adolescents struggled more with mental health and social belonging. The study underscores the need for culturally sensitive and accessible school health services, as well as continued professional development in trauma‐informed care.

### 4.1 Implications

The findings of this study highlight the central role of school nurses in supporting Ukrainian refugee children during their adjustment to life in the new country. School nurses are uniquely positioned to identify early signs of ill health and to promote well‐being through accessible and trust‐based relationships. However, cultural stigma surrounding mental illness created barriers to effective support, as mental health issues were often perceived as taboo and difficult to address openly.

Further, the results suggest a need for ongoing training in cultural competence, including awareness of how cultural norms and taboos may influence help‐seeking and communication. For some school nurses, a lack of knowledge in this area limited their ability to carry out health‐promoting measures.

Future research should explore the perspectives of refugee children themselves, as well as age‐specific challenges identified by school nurses, to inform more equitable and responsive school health services.

## Author Contributions

Study design: Pernille Korf, Pernilla Garmy, and Eva‐Lena Einberg. Data collection: Pernille Korf. Data analysis: Pernille Korf, Pernilla Garmy, and Eva‐Lena Einberg. Study supervision: Pernilla Garmy and Eva‐Lena Einberg. Manuscript writing: Pernille Korf. Critical revisions for important intellectual content: Pernille Korf, Pernilla Garmy, and Eva‐Lena Einberg.

## Funding

The authors have nothing to report.

## Ethics Statement

The study was approved by the Swedish Ethical Review authority before data collection started (2018‐842; 2023‐01448‐02). All methods were carried out in accordance with the human rights principles outlined in the Declaration of Helsinki. Written informed consent was obtained from all participants.

## Conflicts of Interest

The authors declare no conflicts of interest.

## Data Availability

The datasets generated and analyzed during the current study are not publicly available due to protecting the confidentiality of the participants, but are available from the corresponding author on reasonable request.

## Appendix 1 Interview Guide for School Nurses

### 1.1

- What are your experiences of meeting students from Ukraine? Can you tell us about a particular situation?
- Do you have experiences from other periods when refugees came to your school? Is there any difference compared to this time?
- What do students from Ukraine do to feel good?
- How can you tell if students from Ukraine are stressed?
- How can you tell if students from Ukraine are depressed?
- What can students from Ukraine do to feel happier / less depressed? Can you give examples?
- How do you promote well‐being, and reduce stress, loneliness and pain in children and young people from Ukraine?
- Is there anything else related to well‐being and stress for students from Ukraine that you think would be important to discuss?
